# The relevance of cardiac and gastric interoception for disordered eating behavior

**DOI:** 10.1186/s40337-025-01284-0

**Published:** 2025-06-16

**Authors:** Aline Tiemann, Julie Ortmann, Marius Rubo, Andrea H. Meyer, Simone Munsch, Claus Vögele, Zoé van Dyck

**Affiliations:** 1 Department of Clinical Psychology and Psychotherapy, University of Fribourg, Av. de L’Europe 20, 1700 Fribourg, Switzerland; 2 Department of Behavioural and Cognitive Sciences, Institute for Health and Behaviour, University of Luxembourg, 2, place de l’Université, L-4365 Esch-Sur-Alzette, Luxembourg; 3 Department of Cognitive Psychology, Perception and Methodology, Institute of Psychology, University of Bern, Fabrikstrasse 8, 3012 Bern, Switzerland; 4 Department of Psychology, Division of Clinical Psychology and Epidemiology, University of Basel, Missionsstrasse 60/62, 4055 Basel, Switzerland

**Keywords:** Interoception, Cardiac interoception, Gastric interoception, Multidimensional, Disordered eating behavior, Emotional eating, External eating, Restrained eating, Machine learning

## Abstract

**Background:**

Gastric interoception (i.e., the perception of gastrointestinal signals such as hunger, satiety or nausea) in the context of eating has recently gained increasing research attention. Nevertheless, it remains poorly understood how different interoceptive dimensions (e.g., self-report) and organ systems (e.g., cardiac, gastric) relate to each other and to disordered eating behaviors such as emotional, external and restrained eating. We assessed multiple dimensions (behavioral, self-reported, and physiological) in the cardiac domain (interoceptive accuracy, interoceptive self-report, interoceptive insight and objective physiological state) and in the gastric domain (gastric interoceptive sensitivity, gastric attribution of interoceptive sensations, interoceptive self-report, interoceptive insight and objective physiological state). The first goal of this study was to examine the relationship between cardiac and gastric interoception measured via multiple dimensions (behavioral, self-reported, and physiological). The second goal was to investigate whether multidimensional gastric interoception was a more important predictor of emotional, external and restrained eating than multidimensional cardiac interoception.

**Methods:**

Our sample (*n* = 128) was predominantly female (*n* = 116), included healthy individuals (*n* = 87) and individuals with an eating disorder or sub-clinical eating disorder (*n* = 41). Instruments included a heartbeat counting task, the two-step Water Load Test, electrocardiogram, electrogastrogram and the Dutch-Eating Behaviour Questionnaire. We used correlation analysis, multiple regressions, and LASSO regressions. The final sample included in the multiple regression and LASSO regressions resulted in *N* = 89.

**Results:**

Results showed cardiac and gastric interoception to be distinguishable, yet not to be entirely independent processes. Results further suggest gastric and not cardiac interoception to be the most important predictor of emotional, external and restrained eating. Specifically gastric attribution of interoceptive sensations played the most important role in all disordered eating behaviors.

**Conclusions:**

Our findings highlight the importance of the gastric system in the assessment and targeted treatment of disordered eating behaviors. Future research should consider adding additional interoceptive dimensions.

**Supplementary Information:**

The online version contains supplementary material available at 10.1186/s40337-025-01284-0.

## Plain Language Summary

This study examined how awareness of stomach (gastric) and heart (cardiac) signals relates to disordered eating behaviors, such as eating due to emotions, external cues, or restricting food intake. These behaviors increase the risk of eating disorders and are often elevated in those affected. Interoception, which can influence behaviors and emotions, includes multiple aspects, such as sensing and interpreting internal body signals but also the body signal itself. This study aimed to determine whether gastric interoception is more important than cardiac interoception in predicting these eating behaviors.

The study involved 128 participants, mostly women, including healthy individuals and those with eating disorders. Interoception was assessed using tasks like counting heartbeats, drinking water to feel full, and questionnaires about bodily signal perception and eating behaviors.

Results showed gastric and cardiac interoception are distinct but share some links. Awareness of stomach signals was a stronger predictor of disordered eating than awareness of heart signals. Feeling negative after drinking water until full was most strongly associated with disordered eating

These findings emphasize the importance of stomach signal awareness in understanding and treating eating disorders. Future research should explore other aspects of interoception to improve treatments for these conditions.

**Supplementary Information:**

The online version contains supplementary material available at 10.1186/s40337-025-01284-0.

## Background

Disordered eating behaviors pose a problem for both psychological and physical health [1] and increase the risk for the development and maintenance of eating disorders (EDs) [2]. The mechanisms behind disordered eating behaviors, however, remain poorly understood and a better understanding may help to improve future treatment options [2]. In this study, we specifically considered emotional eating (eating in response to emotions), external eating (eating in response to food stimuli regardless of hunger and satiety), and restrained eating (restricting food intake to control weight), which are key behaviors linked to EDs [3, 4], contributing to overeating and binge eating [5]. Altered interoception, which refers to the processing of internal bodily signals, has been suggested as a trans-diagnostic factor underlying disordered eating [2, 6–8].

Interoception refers to a multifaceted system comprising the accurate detection of cardiac (e.g., feeling one’s own heartbeat), gastric, and respiratory signals, pain, or touch, as well as their interpretation and integration by the CNS [9]. In the past, different facets of interoception were used interchangeably, leading to inconsistent findings. Thus, contemporary approaches tend to differentiate between different facets of interoception [10–12]. In this study, we differentiate between *objective physiological states* (such as cardiac or gastric activity) [11], *cardiac interoceptive accuracy* (accurate detection of those physiological states on a behavioral task, such as heartbeat detection tasks) [12], *gastric interoceptive sensitivity* (e.g. sensitivity to gastric signals on a water load task) [13], *interoceptive self-report* (measured via questionnaires or confidence scores and (implicit) prior beliefs believed to influence interoceptive perception in the context of behavioral tasks [10, 12]), *attribution of interoceptive sensations* (interpretation and attributed causality of interoceptive sensations, such as negative affect after ingesting water) [12] and *interoceptive insight* (metacognitive level, i.e., the correspondence between *accuracy* and *self-report*, e.g., via difference scores of heartbeat detection accuracy and confidence) [12]. Interoception is, thus, considered both multidimensional (from physiological signal to detection and interpretation) and multimodal (encompassing multiple organ systems).

The multidimensional assessment of interoception is essential as interoceptive dimensions are distinct and reflect different aspects of interoceptive processes [9, 10, 12]. Specific dimensions may be impaired in certain mental disorders, including EDs [2, 9]. For instance, individuals with anorexia nervosa (AN) may exhibit relatively high interoceptive accuracy/sensitivity but low self-report and insight, struggling to interpret and rely on bodily cues [14]. Recent findings [15] showed that gastric interoceptive self-report (perception of hunger and satiety) varies across ED types: AN patients report lower sensitivity of hunger and higher sensitivity to satiety, while those with bulimia nervosa (BN) and binge-eating disorder (BED) report lower sensitivity to satiety and difficulty distinguishing hunger from emotional states. Differentiating these facets can inform targeted interventions to specific interoceptive impairments [9].

Cardiac interoception is often used as a proxy for general interoception [16], though perceptions across organ systems may be distinct, yet sometimes show significant associations with small effect sizes [13, 17–20]. Prentice and Murphy^21^ concluded that performance in one domain does not predict performance in another, emphasizing the need to differentiate organ systems. In EDs, cardiac and gastric interoception seem to play distinct roles. The link between emotions and bodily signal awareness stems from theories by Lange and James [22], Schachter and Singer [23] and Damasio [24]. Cardiac interoceptive accuracy has been associated with emotional processes [25] with better heartbeat detection linked to stronger emotional experiences [25] and lower interoception associated with alexithymia symptoms [26, 27]. Since emotional processing issues and dysregulation appear to be key aspects in ED development and maintenance [28], cardioception may be linked to ED symptoms [28–30]. Gastric interoception, key for sensing hunger, satiety, and fullness, is thus particularly relevant in ED contexts [2, 7, 8, 13, 31, 32].

In line with this, certain authors propose that cardiac and gastric interoception are distinct [18], as supported by neuroscientific findings [33, 34]. For example, heart-beat evoked potentials (HEP) and gastric-alpha phase-amplitude coupling (PAC), measured via electroencephalogram (EEG), were found to be unrelated, and directing attention to cardiac or gastric signals seems to elicit different neural activation patterns in magnetic resonance imaging (MRI) studies [33, 34]. However, weak correlations between cardiac and gastric indices have been reported [13, 19, 35, 36]. These mixed findings suggest that the relationship between cardiac and gastric interoception is not yet fully understood, necessitating further investigation. The first goal of this study is to explore the relationship between cardiac and gastric interoception using a multidimensional approach. Examining interoception across different levels and organ systems may help to clarify whether it represents a cohesive construct with distinct dimensions, addressing a fundamental premise in interoception research [12].

Interoceptive impairments have been observed across ED diagnoses, subclinical disordered eating behaviors, and post-recovery from EDs [2, 37–39]. Thus, examining specific eating behaviors in different ED samples may offer valuable insights [8, 40]. Emotional eating, external eating, and restrained eating are well-established eating behaviors relevant to EDs [3, 4]. These behaviors contribute to overeating and binge eating [5] and are more pronounced in individuals with EDs compared to healthy controls [41–46]. Restrictive eating, in particular, is prevalent across the ED spectrum [41, 45].

Emotional eating has been negatively associated with *cardiac interoceptive accuracy* (heartbeat counting) and *interoceptive insight* [40, 47]. Additionally, emotional eating has been linked to obesity and overeating [5], and body composition, including body mass index (BMI), weight, and percentage of body fat, may also influence heartbeat detection [48–50], suggesting an interplay between these variables. Findings on self-reported interoceptive abilities are mixed, with some studies identifying associations [47, 51–53] while other did not [40, 54]. Additionally, higher emotional eating levels have been linked to lower *cardiac objective physiological states* (heart rate variability; HRV) [55]. These results suggest that emotional eating correlates with various interoceptive impairments, including reduced cardiac signal detection abilities, poorer metacognitive insight, poorer general and gastric self-reported interoceptive abilities, and lower autonomic self-regulatory abilities.

*Interoceptive self-report* has been found to explain emotional and external eating in overweight children [47]. This suggests that individuals who engage in more external eating often have lower confidence in perceiving their bodily signals and pay less attention to them, potentially due to a learned focus on external rather than internal cues. Dietary restraint was found to be associated with reduced reliance on hunger and satiety cues [39], and higher self-reported interoceptive abilities were linked to lower levels of restrained eating [56]. Impairments in gastric interoception have also been observed in restrained eating, where fullness ratings and shake consumption quantity were not related in individuals with higher levels of restraint [38], consistent with theories suggesting bodily signals to be ignored to restrict food intake [4]. Additionally, heightened HRV has been found to be correlated with increased restrained eating, similar to patterns observed in AN and BN during starvation [57–61].

These findings highlight distinct interoceptive impairments associated with emotional, external, and restrained eating. However, to the best of our knowledge, a systematic investigation into the roles of multidimensional cardiac and gastric interoception in these eating behaviors remains lacking. Understanding whether gastric interoception is more relevant than cardiac interoception in the context of emotional, restrained and external eating requires a comprehensive, multidimensional assessment of both. Most studies have focused on single domains (e.g., cardiac or gastric) and single dimensions (e.g., interoceptive accuracy or self-report) [7, 31, 40, 62, 63]. Gastric interoception has typically solely been assessed via self-report (e.g. interoceptive awareness subscale on the Eating Disorder Inventory; [64]) [2, 31, 39, 52] or behavioral measures (e.g., a water load test) [18, 62, 65]. Thus, the second goal of our study was to determine whether multidimensional gastric interoception better explains emotional, external, and restrained eating than multidimensional cardiac interoception.

### Hypotheses

1. We expect significant associations between measures that reflect the same dimension of interoception (behavioral, self-reported, or physiological) across cardiac and gastric domains [13, 19].
2. We expect gastric interoception to explain emotional, external and restrained eating better than cardiac interoception.

## Methods

### Power analysis, participants and procedure

We used G*Power to conduct an a-priori power analysis. To achieve a medium effect size (*f* = 0.15) in linear multiple regression, with 12 predictors (see below) a sample size of *N* = 127 is required (*α* = 0.05; 1-*β* = 0.80). One hundred and twenty-eight participants (116 females, 12 males) were recruited at the University of Luxembourg through flyers, university e-mail lists, posters and local newspapers. Due to missing data, eighty-nine participants were included in the final sample for multiple regression and least absolute shrinkage and selection operator (LASSO) analysis. The data for the current study was collected as part of two studies with the same design and instruments. One of the studies specifically targeted the recruitment of individuals with binge-eating episodes. The two studies where combined to increase statistical power through a larger sample size and to cover a broad spectrum of eating behavior. Participants were required to be at least 18 years of age and both individuals with and without ED symptoms were invited to participate. Exclusion criteria concerned gastrointestinal surgeries, chronic and/or current physical illnesses, as well as any treatment or condition strongly affecting weight or eating behavior, current or past psychotic disorders, bipolar disorder or suicidal ideation. The sample included 38 participants who met DSM-5 criteria for EDs, namely current BN (*n* = 13) and BED (*n* = 25). The sample also included sub-clinical BED (*n* = 3). The average illness duration was 26.9 months (*SD* = 13.55), 13 individuals had received treatment in the past, and four reported successful treatment completion. ED diagnoses were assessed through the Eating Disorder Examination (EDE) [66, 67] and the Structured Clinical Interview for DSM-IV Axis I (SCID) [68, 69]. Ethical approval was obtained from the Ethics Review Panel of the University of Luxembourg (substudy 1: ERP 22-017 A EDIP). Before data collection commenced, written informed consent was provided by all participants and their participation was compensated with 80€ and 60€, depending on the study. Participants were asked to fast at least 3 h before and not to consume any beverages for at least 2 h before taking part in the study. Participants first completed diagnostic interviews and questionnaires. This was followed by height and weight measurements. Next, a 5-min resting electrocardiogram (ECG) recording was conducted, followed by a heartbeat counting task [70]. Participants then underwent the two-step Water Load Test (WLT-II) [13] and completed the procedure with a 15-min resting electrogastrogram (EGG) recording. The WLT-II was carried out at the end, as fasting states may affect heartbeat perception [71].

### Body mass index (BMI)

Participants were weighed and measured during laboratory testing to calculate their BMI as follows: *kg/m*^*2*^. The study used calibrated scales.

### Cardiac interoception

#### Cardiac interoceptive accuracy: heartbeat counting task (HBCT)

A heartbeat counting task (HBCT) [70] was used to measure cardiac *interoceptive accuracy*. Participants were asked to silently count their heartbeats without measuring their pulse during four time intervals (25°s, 35°s, 45°s and 55°s, randomized order). During the entire task, ECG was recorded at a sampling rate of 1000 Hz with a BIOPACTM MP150 (Biopac Systems Inc., USA) and processed with the software Acqknowledge 4.2. Cardiac *interoceptive accuracy* was then calculated using the formula:

$$\raise.5ex\hbox{$\scriptstyle 1$}\kern-.1em/ \kern-.15em\lower.25ex\hbox{$\scriptstyle 4$} \left( {1 - \left( {|{\text{actual heartbeats }}{-}{\text{ reported heartbeats}}|} \right)/\left( {\text{actual heartbeats}} \right)} \right)$$

The average of the four trials resulted in a mean cardiac *interoceptive accuracy* score for each participant (ranging between 0 and 1), with higher scores corresponding to a higher cardiac interoceptive accuracy.

#### Cardiac interoceptive self-report: confidence ratings on the HBCT

To measure cardiac *interoceptive self-report*, after each trial, participants were asked to indicate their confidence in their performance on the HBCT on a Likert scale of 0 “not sure at all” to 8 “absolutely sure”. Higher mean scores indicate a higher self-reported cardiac *interoceptive self-report*, i.e., confidence in one’s interoceptive ability during the HBCT.

#### Cardiac interoceptive insight

Cardiac *interoceptive insight* concerns the correspondence between cardiac *interoceptive accuracy* and *self-report* [10]. Higher *interoceptive insight* corresponds to higher insight into one’s interoceptive abilities [10]. The correspondence between interoceptive accuracy and self-report can be operationalized via correlations [10], though this comes with certain drawbacks. For instance, if a participant reports the same confidence level across all trials, no correlation can be calculated due to a lack of variance [72]. Additionally, intraindividual correlations reflect the covariation between confidence and performance but ignore the absolute discrepancy between them. For example, a participant with low interoceptive accuracy scores (“0.1”, “0.2”, “0.3”, “0.4”) and high confidence ratings (“5”, “6”, “7”, “8”) may achieve a perfect correlation of 1, indicating perfect interoceptive insight despite a significant mismatch between performance (mean of 0.25) and confidence (mean of 6.5). Due to these limitations, we propose a different method for calculating interoceptive insight, absolute difference scores. More specifically, to calculate *interoceptive insight*, we used the “Percent of Maximum Possible” (POMP) scoring method [73], which has also been used by Weineck and colleagues^74^. POMP-Scores are calculated with the following formula:

$${\text{POMP}}\, = \,\left( {\left( {x{-}{\text{variable minimum score}}} \right)/\left( {{\text{maximum}}{-}{\text{minimum }}score} \right)} \right) \, \times \, 100.$$

This results in scores ranging from 0 to 100. POMP-Scores prevent problems arising with z-standardization [75]. To calculate cardiac *interoceptive insight*, cardiac *interoceptive accuracy* and *self-report* were first converted into POMP-scores. Then, absolute difference scores [76] between the *accuracy* and *self-report* POMP-scores were calculated (|CIAw_POMP_ = CISP_OMP_$-$ ICA_ccPOMP_|) (note: CIAw_POMP_ = cardiac interoceptive insight, CIS_POMP_ = cardiac *interoceptive self-report,* CA_ccPOMP_ = cardiac *interoceptive accuracy)*. The resulting CIAw score was then subtracted from 100 [74]. This results in more intuitive values, as higher scores correspond to a higher insight.

#### Cardiac objective physiological state: electrocardiogram

A five-minute resting ECG was recorded for every participant. Beforehand, participant’s skin was cleaned with alcohol and three disposable electrodes (Ag/AgCl) were placed on the participant’s chest in accordance with the “Einthoven Triangle” [77]. The positive and ground electrodes were placed on the right and left costal margin on either side of the abdomen, while the negative electrode was placed beneath the collar bone. Heart rate (HR) and HRV were analyzed with the software “ARTiiFACT” [78]. Through linear interpolation, artefacts of the “inter-beat-intervals” (IBIs) were removed. Two measures were extracted after Fast Fourier Transformation (FFT) of the RR-interval series: [1] the root mean square of successive differences (RMSSD) and [2] high frequency normalized units (HFnu). RMSSD is a time domain measure, while HFnu is a frequency domain measure. RMSSD and HFnu are highly correlated and have been suggested to represent indices of the parasympathetic nervous system on HRV [79–81]. RMSSD has been proposed to also reflect sympathetic activation [81].

### Gastric Interoception

#### Gastric interoceptive sensitivity: two-step water load test (WLT-II)

Gastric *interoceptive sensitivity* was measured using the WLT-II [13]*.* Participants drank non-carbonated water at room temperature from a non-transparent 5-L flask using a long straw to regulate swallowing size. The flask was filled with 1.5 L of water, unbeknownst to the participants, to ensure their safety by not exceeding the 1.5-L maximum, blind them to the actual amount consumed, and give an impression of unlimited water supply.

There were two 5-min drinking phases: until satiation and until maximum fullness. Participants were not informed about the second drinking phase beforehand. In the first phase, they were instructed to drink water until the first sign of satiation that corresponds to meal termination. During the second phase, participants were instructed to drink water until they reached the maximum point of stomach fullness. Each drinking phase was followed by a 10-min rest period, giving the experimenter the time to record the amount of consumed water. Indices that were calculated were: ingested water volume until satiety, additional water volume until maximum fullness, the total ingested water volume and the percentage of satiation to maximum fullness.

#### Gastric interoceptive self-report and gastric attribution of interoceptive sensations (WLT-II questionnaire)

Gastric *interoceptive self-report* and *gastric attribution of interoceptive sensations* were assessed with the WLT-II questionnaire [13]. This questionnaire is used to measures sensations related to water ingestion. Participants are asked to focus on their current abdominal sensations and rate their current satiation, fullness and negative affect before (t0) and after the first drinking phase (t1) as well as after the second drinking phase (t2). We used satiation and fullness ratings for *gastric interoceptive self-report* and the negative affect scale for *gastric attribution of interoceptive sensations.* The negative affect (NA) scale consists of five items (guilt, nausea, discomfort, sluggishness, and arousal) on a 7 point-Likert scale (1 = no sensation/not at all, 7 = extremely). In the present study, WLT-II questionnaire ratings after water ingestion (t2) will be analyzed. In the current study, the NA scale showed acceptable internal consistency with α = 0.79, which is comparable to previous research [13] with α = 0.81.

#### Gastric interoceptive insight

Gastric *interoceptive insight* was calculated using the same method as cardiac *interoceptive insight* (see Sect. 2.3.3). For gastric *interoceptive accuracy* (in our case *gastric interoceptive sensitivity)*, the percentage of satiation to maximum fullness from the WLT-II was used. For gastric *interoceptive self-report*, the corresponding satiation rating from the WLT-II questionnaire was taken. Both were converted into POMP-Scores, their absolute difference was calculated and subtracted from 100. This resulted in gastric *interoceptive insight* scores, where higher scores reflect higher *insight*.

#### Gastric objective physiological state: electrogastrography (EGG)

Gastric activity is involved in the regulation of the development of satiation and in digestion and can be recorded using EGG [82]. To consider the fourth dimension of gastric interoception [11] (i.e., *objective physiological states*), gastric activity can be analyzed. In healthy individuals, the stomach usually contracts approximately three times per minute (3 cycles per minute; cpm) [83]. Ingesting water increases the amount of normal 3 cpm gastric activity (which is called normogastria) in healthy participants [83]. Gastric myoelectrical activity was recorded during a 15-min resting phase before the first drinking phase of the WLT-II. Then, after the two drinking phases, another 15 min EGG was monitored. To enhance the transmission of the signal, participant’s skin was cleaned with alcohol and gently abraded (Nuprep, D.O. Weaver and Co., Aurora, CO). Three abdominal electrodes (ConMed Cleartrace) were placed over the region of the gastric antrum [83]. The first active electrode was positioned between the midpoint of the umbilicus and the xiphoid notch, while the second one was placed approximately 6 cm left of the abdominal midline. The reference electrode was attached approximately 10 cm to the right from the midline electrode. For the EGG recording, participants sat in a half-reclining, comfortable chair at approximately 30–45°. They were asked to not to talk and minimize movement during the recording.

EGG data was recoded on a hard disk using two different amplifier systems: the BrainAmp ExG amplifier by Brain Products, Gilching, Germany, which sampled the data at 5000 Hz, and the Biopac MP150 amplifier system by Biopac Systems, Inc., which had an EGG100 C module for EGG assessment at 16-bit resolution and a sampling rate of 1 kHz. A hardware low-pass filter with a cut-off frequency of 1000 Hz and no high-pass filter (DC recording) was used.

Data analysis was carried out using WinCPRS 1.160 software (Absolute Aliens Oy, Turku, Finland). The analysis was performed separately for both 15 min segments. The data was visually inspected to determine general quality and possible artifacts. Only continuous recordings without artifacts and visually identifiable waveforms were included. The data was software-filtered (0.016–0.25 Hz, i.e., 1–15 cpm) [83], down-sampled (to 10 Hz), linearly interpolated, tapered with a Hanning window as well as zero padded to the next power of 2. Then, to extract the different frequency power bands, a FFT was performed with runs of 240 s and a 75% overlap [83]. The following power bands were extracted: bradygastria (1.0–2.5 cpm), normogastria (2.5–3.75 cpm) and tachygastria (3.75–10.0 cpm). The power in each EGG band was calculated for the second time point (t2) as a percentage of the total EGG band power within the corresponding frequency range.

### Dutch eating behaviour questionnaire (DEBQ)

To assess emotional, external and restrained eating, the German version of the DEBQ was employed. The German DEBQ [3] consists of 30 items with three sub-scales which play a role in EDs and obesity [2]: Emotional Eating (10 items), External Eating (10 items) and Restrained Eating (10 items). Emotional Eating corresponds to eating in response to diffuse or clearly labelled emotions, usually to negative emotional content (e.g., “*Do you have the desire to eat when you are emotionally upset?*”). External Eating refers to eating in response to external food stimuli (e.g., “*Do you eat more than usual, when others are eating*?”). Restrained Eating corresponds to restricting food intake for the purpose of weight control (e.g., “*Do you take into account your weight with what you eat*”). Response options are on a 5-point Likert-Scale (1 = “*never*”, 3 = “*sometimes*”, 5 = “*always*”). To obtain sub-scale scores, item scores are averaged, with higher mean scores corresponding to higher tendencies of each eating behavior. Each sub-scale had high internal consistency. Emotional Eating (Cronbach’s α = 0.94 in [4], vs α = 0.96 in the present sample), External Eating (α = 0.80 vs. α = 0.73), Restrained Eating (α = 0.95, vs. α = 0.88). The total questionnaire showed good internal consistency with an α = 0.92, which is comparable α = 0.94 in the original study.

### Data analysis

For Hypothesis 1, Spearman correlations were calculated due to multiple non-normally distributed variables. We tested normality through visual (Q-Q plots, histograms, box plots) and statistical inspection (Kolmogorov–Smirnov and Shapiro–Wilk tests). For Hypothesis 2, we first examined Spearman correlations between the predictors and outcome variables, before we compared two modeling approaches [1] standard multiple regression and [2] LASSO regression in their prediction performance. Both approaches were cross validated. Cardiac and gastric interoceptive dimensions were used as predictors and emotional, external and restrained eating as outcome variables. We tested the assumptions of multiple linear regression with the usual methods. Due to multicollinearity and aliasing issues, five variables with a variance inflation factor (VIF) over 10 were excluded and only one variable of interest per interoceptive dimension was retained in these cases [84]. This means that for the WLT -II indices, the percentage of satiation to maximum fullness was retained, while the following variables were excluded: ingested water volume until satiety, additional water volume until maximum fullness, the total ingested water volume. For the gastric objective physiological state, only normogastria was retained, while bradygastria and tachygastria were excluded. For the final regression analyses, the following 12 variables were used (see Table 1):

**Table 1:** Overview of retained variables for multiple linear regression and LASSO regression after adjusting for multicollinearity

	Cardiac	Gastric
Cardiac interoceptive accuracy/gastric interoceptive sensitivity	HBCT scores	Percentage of satiation to maximum fullness on the WLT-II
Interoceptive self-report	HBCT confidence ratings	Satiation and fullness of WLT-II questionnaire
Gastric attribution of interoceptive sensations		Negative affect (NA) of WLT-II questionnaire
Interoceptive insight	Cardiac interoceptive insight	Gastric interoceptive insight
Objective physiological state	Mean HR, HRV (RMSSD), HRV (high frequency normalized units)	Normogastria

LASSO regression was used as an additional analysis, as multiple regression often results in overfitting [85], leading to low predictive accuracy. This can especially be the case when the number of predictors is relatively high compared to a rather small sample, such as in the present study [86]. LASSO performs variable selection; it shrinks regression coefficients of less important variables to 0. This can help generating models that are more parsimonious by discarding less important variables, and thus enable to build more robust predictive models with better predictive accuracy. As required by LASSO regression, we omitted missing values by listwise deletion and, thus, our final sample size for regression analyses consisted of *n* = 89. The following data was missing: questionnaire data (BMI *n* = 5, DEBQ *n* = 8), behavioral data (cardiac interoceptive accuracy *n* = 15, cardiac interoceptive self-report *n* = 11, gastric interoceptive self-report *n* = 19, gastric interoceptive self-report *n* = 11) and physiological data (ECG-parameters *n* = 12, EGG parameters *n* = 19). As cardiac and gastric interoceptive insight is calculated from interoceptive accuracy and self-report, missing data results as a sum of their missing data, resulting in *n* = 15 and *n* = 20, respectively. From the missing data, 9 individuals were dropouts with only questionnaire data, but no laboratory tasks data. The remaining missing data (excluding dropouts) for the WLT-II, HBCT and EGG- and ECG-parameters were due to technical issues during the testing sessions. For the EGG analysis, missing data in our dataset are attributable to recordings compromised by noise, artifacts, or poor signal quality.

Statistical analyses were carried out with IBM SPSS Statistics Version 26 (SPSS Inc. Chicago, IL) and R version 4.2.2 [87] for Windows 10. Cross-validated multiple regression and LASSO regression were estimated using the *caret* package version 6.0.93 [88]. Variables were standardized to a mean of zero and a standard deviation of 1 to facilitate the interpretation of regression coefficients and obtain more interpretable and stable results [85]. For both regression types, a tenfold cross-validation was used, meaning that the data was randomly partitioned into 10 equally sized subsets. The models were trained on the 9 subsets and tested on the remaining subset, with 10 repetitions. Predictive performance was evaluated based on [1] *R* squared (*R*^*2*^), corresponding to the percentage of explained variance of the outcome variable by the model, with higher percentage indicating better model fit, on [2] Mean Absolute Error (*MAE*), reflecting the mean difference between predicted and actual values, with smaller values indicating better model fit and on [3] the Root Mean Square Error (*RMSE*), being similar to *MAE* but taking into account direction of error due to squaring of the differences between predicted and actual values (lower values also indicate better model fit). The significance level was set to *p* < 0.05.

## Results

### Demographic characteristics

Demographic characteristics can be found in Table 2.

**Table 2:** Socio-demographic characteristics of participants

	HP (n = 87)	ED total (n = 41)	BE (n = 28)	BN (n = 13)	Total (n = 128)	Difference HP and ED
*Sex*						
Female	79 (90.6%)	37 (90.2%)	23 (82.1%)	12 (92.3%)	116 (90.6%)	
Male	8 (9.4%)	4 (9.8%)	3 (17.9%)	1 (7.7%)	12 (9.4%)	
*Age range*						
18–30	39	17	10	6	56	
31–40	16	9	5	4	25	
41–50	14	7	5	3	21	
50 +	18	8	8	0	26	
*Age*						
M (SD)	36.24_a_ (13.29)	37.20 (12.23)	40.03_a_ (13.15)	32.86_a_ (8.02)	36.73 (12.91)	*p* = 0.210, η^2^ = 0.02
*BMI*						
M (SD)	27.47_a_ (6.96)	36.64 (12.93)	33.19_b_ (7.71)	26.09_a_ (6.62)	28.50 (7.43)	*p* = 0.001, η^2^ = 0.10

*Note.* *BE* Binge Eating Group, including binge-eating disorder (BED) and sub-clinical BED. *BMI* Body Mass Index. *BN* Bulimia Nervosa. *ED* Eating Disorder. *HP* healthy participants. *M* Mean. *SD* Standard Deviation

### Descriptives

Descriptive statistics of the variables included in the analyses can be found in Table 3. Values were comparable to previous studies regarding the HBCT [13, 19], ECG [89], WLT-II [13, 82] and EGG after the WLT-II [82]. For the DEBQ, our sample was comparable to previous studies for the healthy individuals (emotional eating *M* = 2.44, *SD* = 0.97, external eating *M* = 3.04, *SD* = 0.55, restrained eating *M* = 2.72, *SD* = 0.75) and bulimic patients (emotional eating *M* = 3.75, *SD* = 0.75, external eating *M* = 3.43, *SD* = 0.55, restrained eating *M* = 3.08, *SD* = 0.82) [3, 46].

**Table 3:** Descriptive statistics of the different dimensions of cardiac and gastric interoception and emotional, external and restrained eating

	*n*	*M*	*SD*	*Min*	*Max*
*Cardiac interoceptive accuracy*	113	0.59	0.25	0	0.98
*Cardiac interoceptive self-report*	117	3.94	2.06	0	8
*Cardiac interoceptive insight*	112	74.57	21.59	0	100
*Cardiac objective physiological state*					
Mean HR	116	67.62	11.10	44	99.22
HRV(RMSSD)	116	43.07	28.70	6.33	143.21
HRV (HFnu)	166	50.92	21.40	8.79	88.70
*Gastric interoceptive sensitivity*					
Ingested water volume until satiety (ml)	109	453.65	212.414	102	1210
additional water volume until maximum fullness (ml)	109	367.63	213.28	12	1498
total ingested water volume (ml)	109	821.28	344.40	169	1970
percentage of satiation to maximum fullness	109	56.03	14.31	23.96	94.09
*Gastric Interoceptive self-report*					
Satiation	117	5.80	1.52	1	7
Fullness	117	5.79	1.59	1	7
*Gastric attribution of interoceptive sensations*					
Negative affect	117	2.88	1.19	1	6.2
*Gastric interoceptive insight*	108	75.09	17.11	23.36	99.74
*Gastric objective physiological state*					
Bradygastria (%)	109	41.20	14.89	12.01	84.35
Normogastria (%)	109	34.86	15.46	12.09	81.34
Tachygastria (%)	109	19.68	9.91	2.83	52.63
*DEBQ*					
DEBQ emotional eating	120	2.86	1.08	1.00	5
DEBQ external eating	120	3.16	0.58	1.50	4.70
DEBQ restrained eating	120	2.84	0.79	1.30	4.60

### Hypothesis 1: correlations between the same dimensions of cardiac and gastric interoception

For the complete correlation matrix, see Table 7 in supplement. Cardiac interoceptive accuracy did not correlate with gastric interoceptive sensitivity: ingested water volume until satiety (ml) (*r* = 0.12, *p* = 0.216), additional water volume until maximum fullness (ml) (*r* = 0.14, *p* = 0.144), total ingested water volume (ml) (*r* = 0.17, *p* = 0.09) or the percentage of satiation to maximum fullness (*r* = −0.05, *p* = 0.651). Cardiac interoceptive self-report did not correlate significantly with gastric interoceptive self-report (satiation (*r* = −0.08, *p* = 0.422), fullness (*r* = −0.04, *p* = 0.672)) nor with gastric attribution of interoceptive sensations (negative affect (*r* = −0.11, *p* = 0.245)). Cardiac interoceptive insight did not significantly correlate with gastric interoceptive insight (*r* = −0.02, *p* = 0.82). Mean heart rate (HR) did not correlate significantly with bradygastria (*r* = 0.18, *p* = 0.067), normogastria (*r* = 0.05, *p* = 0.607), or tachygastria (*r* = −0.12, *p* = 0.232). HRV (RMSSD) showed a significant negative correlation with bradygastria (*r* = −0.20, *p* = 0.042), but did not correlate significantly with normogastria (*r* = 0.03, *p* = 0.727) or tachygastria (*r* = 0.13, *p* = 0.201). HRV (HFnu) did not correlate significantly with bradygastria (*r* = −0.03, *p* = 0.795), normogastria (*r* = 0.05, *p* = 0.587), or tachygastria (*r* = 0.00, *p* = 0.997).

### Hypothesis 2: explaining emotional, external and restrained eating from cardiac and gastric interoception

The correlation matrix for all relevant variables can be found in Tables 8 and 9 in the supplements. In our statistical analyses with emotional, external and restrained eating, we noticed that controlling for BMI did not have an impact on our results. We, therefore, report the present results without controlling for BMI. However, there were significant correlations between BMI and emotional eating (*r* = 0.35, *p* < 0.001), external eating (*r* = 0.20, *p* = 0.032) and restrained eating (*r* = 0.20, *p* = 0.035). There were significant positive correlations between BMI and fullness (*r* = 0.19, *p* = 0.042) and a significant negative correlation between BMI and normogastria (*r* = – 0.23, *p* = 0.019). We did, however, control for sex by adding it as a covariate.

#### Outcome: emotional eating

##### Multiple regression

Coefficients of the multiple linear regression model can be found in Table 4. The model significantly explained emotional eating (*F*(13, 75) = 2.75, *p* = 0.003). NA was a significant predictor of emotional eating, while fullness, normogastria and cardiac *interoceptive accuracy* were marginally significant.

**Table 4:** Results of multiple linear regression and lasso regression with emotional eating as outcome variable

	Multiple linear regression model				LASSO
	β*(n* = 89)	Std. error	*t* -value	*p*	β
Fullness	0.24	0.14	1.700	0.093	0.21
Negative affect	0.23	0.11	1.997	0.049*	0.20
Normogastria	– 0.19	0.10	– 1.844	0.069	– 0.14
Cardiac interoceptive insight	0.14	0.10	1.363	0.177	0.11
HRV(HFnu)	– 0.17	0.11	– 1.630	0.107	– 0.10
Mean HR	– 0.20	0.12	– 1.642	0.105	– 0.10
Cardiac interoceptive accuracy	– 0.18	0.10	– 1.753	0.084	– 0.10
Sex	– 0.11	0.11	– 1.002	0.320	– 0.07
Satiation	0.03	0.13	0.208	0.836	0.0005
Cardiac interoceptive self-report	0.06	0.11	0.591	0.557	0.00
HRV(RMSSD)	– 0.04	0.12	– 0.305	0.761	0.00
Percentage of satiation to maximum fullness	– 0.01	0.10	– 0.051	0.959	0.00
Gastric interoceptive insight	0.00	0.10	– 0.011	0.991	0.00

*p < 0.05

##### LASSO regression

The LASSO regression selected 8 out of 12 predictor variables to explain emotional eating. The most important predictors were fullness and NA, followed by normogastria, cardiac *interoceptive insight*, HRV (HFnu), mean HR and *cardiac interoceptive accuracy* and satiation (see Table 4). LASSO regression (*R*^*2*^ = 0.227, *RMSE* = 0.917, *MAE* = 0.780) outperformed multiple regression after cross-validation (*R*^*2*^ = 0.235, *RMSE* = 0.953, *MAE* = 0.791) by demonstrating better predictive value based on *MAE* and *RMSE*.

#### Outcome: external eating

##### Multiple regression

Coefficients of the multiple linear regression model can be found in Table 5. The model did not significantly explain external eating (*F* (13, 75) = 1.53, p = 0.130).

**Table 5:** Results of multiple linear regression and lasso regression with external eating as outcome variable

	Mutiple linear regression model				LASSO
	β *(n* = 89)	Std. error	*t* -value	*p*	β
Negative affect	0.38	0.12	3.113	0.003**	0.16
Sex (covariate)	0.10	0.12	0.831	0.409	0.006
Cardiac interoceptive self-report	0.14	0.11	1.230	0.223	0.00
Cardiac interoceptive accuracy	0.03	0.11	0.253	0.801	0.00
Cardiac interoceptive insight	0.13	0.11	1.145	0.256	0.00
Mean HR	0.07	0.13	0.520	0.604	0.00
HRV(RMSSD)	0.16	0.13	1.287	0.202	0.00
HRV(HFnu)	– 0.24	0.12	– 2.118	0.038*	0.00
Normogastria	0.10	0.11	0.907	0.367	0.00
Percentage of satiation to maximum fullness	0.12	0.11	1.154	0.252	0.00
Satiation	– 0.19	0.14	−1.408	0.163	0.00
Fullness	0.11	0.15	0.724	0.471	0.00
Gastric interoceptive insight	– 0.03	0.11	−0.266	0.791	0.00

**p < 0.01

##### LASSO regression

The LASSO regression selected 1 out of 12 predictor variables to explain external eating. The only important predictor was NA (see Table 5). LASSO regression (*R*^*2*^ = 0.190, *RMSE* = 0.984, *MAE* = 0.746) showed better predictive value, based on *R*^*2*^ and *RMSE* and *MAE*, than the multiple regression after cross-validation (*R*^*2*^ = 0.163, *RMSE* = 1.076, *MAE* = 0.830).

#### Outcome: restrained eating

##### Multiple regression

Coefficients of the multiple linear regression models can be found in Table 6. The model significantly explained restrained eating *F *(13, 75) = 1.94, p = 0.038). NA significantly explained restrained eating.

**Table 6:** Results of multiple linear regression and lasso regression with restrained eating as outcome variable

	Multiple linear regression				LASSO
	β *(n* = 89)	Std. error	t -value	*p*	β
Negative affect	0.33	0.12	2.735	0.008	0.21
Sex (covariate)	– 0.19	0.12	– 1.579	0.119	– 0.21
Mean HR	– 0.19	0.13	– 1.498	0.138	– 0.13
HRV(RMSSD)	0.15	0.12	1.229	0.223	0.07
Cardiac interoceptive self-report	0.02	0.11	0.218	0.828	0.00
Cardiac interoceptive accuracy	0.07	0.11	0.652	0.516	0.00
Cardiac interoceptive insight	0.01	0.11	0.101	0.920	0.00
HRV(HFnu)	– 0.05	0.11	– 0.505	0.615	0.00
Normogastria	– 0.04	0.10	– 0.352	0.726	0.00
Percentage of satiation to maximum fullness	0.10	0.13	0.779	0.438	0.00
Satiation	– 0.11	0.15	– 0.736	0.464	0.00
Fullness	0.03	0.10	0.289	0.773	0.00
Gastric interoceptive insight	0.33	0.12	2.735	0.008	0.00

##### LASSO regression

The LASSO regression selected 3 out of 12 predictor variables to explain restrained eating. The most important predictors were NA, mean HR and HRV(RMSSD (see Table 6). LASSO regression (*R*^*2*^ = 0.235, *RMSE* = 0.930, *MAE* = 0.790) showed better predictive value, based on *R*^*2*^ and *RMSE*, than the multiple regression after cross-validation (*R*^*2*^ = 0.154, *RMSE* = 1.047, *MAE* = 0.880).

## Discussion

The first goal of our study was to examine the association between cardiac and gastric interoception across the same dimensions (behavioral, self-reported, or physiological). We found no significant associations between cardiac accuracy and gastric sensitivity at the behavioral level. Similarly, there were no associations between cardiac self-report and gastric self-report or gastric attribution of interoceptive sensations (self-report level), nor between cardiac and gastric interoceptive insight. A small negative association was observed between cardiac and gastric objective physiological states. For the second goal of our paper, we expected multidimensional gastric interoception to be a better predictor for emotional, external and restrained eating than multidimensional cardiac interoception, which was confirmed by our results.

We did not find an association between *cardiac interoceptive accuracy* and *gastric interoceptive sensitivity* indicating their distinctiveness. These results are in line with certain previous findings [18], but differ from other studies which found [13, 19, 35, 36]. For associations between *interoceptive accuracy/sensitivity* measures across other organ systems, such as respiratory and cardiac interoception, results are even more inconclusive [17, 20, 90]. The heterogeneity of results may stem from the fact that accuracy and sensitivity, although considered to be equivalent in the past, actually measure different processes. Indeed, while cardiac interoceptive accuracy reflects the “correspondence between objectively measured physiological events and individual’s reported experience of those events” [12], gastric interoceptive sensitivity, as measured by water load tests, primarily captures subjective sensitivity to gastric sensations (e.g., satiety and fullness), which is more difficult to link to objective physiological events (e.g., there is no objective satiety or fullness threshold). Future research should focus on developing tasks that measure comparable interoceptive accuracy/sensitivity capabilities in the cardiac and gastric domain. We did not find an association between cardiac and gastric *interoceptive self-report/attribution of interoceptive sensations,* suggesting that one type of *interoceptive self-report* (e.g., cardiac or gastric) may not necessarily be related to another. This aligns with recent findings [52], observing no significant link between general self-reported interoception and self-reported hunger and satiety responsiveness. For *interoceptive insight*, there were no significant associations. To our knowledge, there are no studies yet comparing cardiac and gastric *interoceptive insight*. For *objective physiological state*, we found a small negative association between cardiac and gastric indices (HRV RMSSD and bradygastria), which suggests that lower levels of HRV are associated with higher levels of bradygastria after water ingestion. This may be attributed to the involvement of both vagal and sympathetic pathways in HRV and gastric activity [91]. Our findings suggest that cardiac and gastric interoception are distinguishable, yet not entirely separate processes, operating independently while sharing certain biological pathways and abilities [18]. These processes warrant individual examination, which aligns with recent neuroscience research, according to which cardiac and gastric interoception are associated with distinct neural processes [33, 34]. Overall, these results underscore the importance of distinguishing both organ systems and interoceptive dimensions. They also cast doubt on generalizing interoceptive capacities from one organ system to another and across dimensions [12].

For emotional eating, we found gastric interoception to be a better predictor than cardiac interoception consistently across both the multiple regression and the LASSO regression. Multiple regression identified *gastric attribution of interoceptive sensations* (negative affect after water consumption) as the strongest predictor of emotional eating, followed by marginally significant predictors: gastric fullness ratings, normogastria, and cardiac interoceptive accuracy. Gastric attribution of interoceptive sensations (negative affect), gastric interoceptive self-report (fullness ratings) and normogastria turned out to be key predictors but additional factors, including cardiac interoceptive insight, HRV (HFnu), mean HR, cardiac accuracy, and gastric satiation ratings are also of importance. In more detail, emotional eating was associated with higher levels of water ingestion until satiation, accompanied with corresponding higher levels of self-reported satiation, fullness and negative affect after water ingestion, as well as lower levels of the percentage of normogastria. This suggests that individuals with higher levels of emotional eating perceive their comfortable satiety and fullness thresholds less accurately, and, thus, drank beyond these. This was also mirrored in the EGG, where higher levels of emotional eating were associated with lower levels of normogastria after water ingestion. It is plausible that higher water ingestion led to nausea, which reduced normogastria [83]. Indeed, we found higher water ingestion until satiation to be positively correlated with nausea (*r* = 0.29, *p* = 0.013), and negatively with normogastria (*r* = – 0.20, *p* = 0.046). Individuals with high emotional eating scores might notice their gastric sensations “too late”, aligning with patterns of losing control during eating [5]. In other words, they may lack a fine-grained perception of their satiety and fullness thresholds, leading to overconsumption. Another explanation could be that they display impairments in predictive coding (i.e., anticipation of future visceral sensations, based on previous experience and taking into account the current experience) [6, 32].

Although gastric interoception seems to play the lead role in emotional eating, the current results suggest that cardiac interoception is important as well – albeit to a smaller extent. This fits previous findings in the field [40, 51, 56]. Cardiac and gastric interoception are both recognized as important in emotional processing, making their involvement in emotional eating logically consistent [92, 93]. Gastric interoception is thought to play a role in food-related decision-making processes, as stomach distention activates vagal afferent neurons, which transmit information about stomach volume changes to the brain [94]. Cardiac interoception, on the other hand, is often considered as a measure of overall interoceptive abilities and has been associated with experienced hunger and intuitive eating styles [95, 96]. In the current sample, emotional eating was associated with lower levels of HRV (HFnu), mean HR, and lower heartbeat detection accuracy, and corresponding higher levels of cardiac metacognitive insight. Enhanced cardiac interoception has been linked to more adaptive emotion regulation [97], indicating that in the context of emotional eating, which arguably represents a maladaptive attempt of emotion regulation, it follows logically that lower cardiac interoceptive accuracy (and corresponding lower levels of HRV and mean HR) shows associations with higher level of emotional eating. Indeed, studies have shown that a range of negative emotions can increase the vulnerability to emotional eating, including depressive symptoms, anxiety/anger, boredom, shame but also, although to a lesser extent, positive emotions [98–102]. However, it seems that metacognitive insight of a person’s detection ability does not follow the same pattern, indicating once more that these dimensions are distinct and capture separate processes. BMI, which positively correlated with emotional eating in the present study, might also play a role in the relationship between emotional eating and cardiac interoception. While not significantly associated in the present study, previous studies have shown that a higher BMI is associated with lower heartbeat detection and HRV [48–50, 103], suggesting a possible mediating role of body composition in the relationship between cardiac interoception and emotional eating.

For external eating, gastric interoception also emerged as a more important factor than cardiac interoception. The multiple regression analyses did not yield significant results, and the LASSO regression only selected *gastric attribution of interoceptive sensations* (negative affect after water ingestion) as a predictor. When looking at the correlations, higher scores in external eating were associated with higher levels of ingested water volume until satiety, higher subjective fullness, and higher negative affect ratings after drinking water. Individuals more inclined towards external eating may have higher satiety thresholds, leading to greater fullness and more discomfort after drinking water. This aligns with theories and neuroscientific findings of external eating, suggesting that interoceptive cues are less considered in food consumption decisions, with individuals relying more strongly on external cues than on internal ones to regulate hunger and satiety [4, 104–106]. Another hypothesis is that individuals who score high on external eating—just as individuals scoring high on emotional eating – fail to integrate internal signals into the food-based decision making process due to prediction errors (i.e., failure to anticipate their future bodily states) and thus have to rely on external cues to gather information about their body and infer their internal state (e.g., body checking, portion size…) [8].

The multiple regression model significantly explained restrained eating and found *gastric attribution of interoceptive sensations* (negative affect) to be a significant predictor. The LASSO regression selected three predictors: *gastric attribution of interoceptive sensations* (negative affect) and cardiac *objective physiological state* (HRV and HR), which is consistent with previous findings [57, 59, 61]. Lower HR and higher HRV in restrictive eaters could result from increased HRV following fasting, which has been found in both healthy individuals and individuals with BN [60, 71]. Changes in HRV may be due to alterations in cardio-dynamic parameters induced by fasting (i.e., heightened cardiac sympathetic activity), which has been linked to increased *interoceptive accuracy* when experimentally induced through physical exercise or body tilting (i.e., putting the body at a different angle via a tilt-table) [71, 107]. These LASSO regression findings complete the regression analysis, showing that both gastric and cardiac interoception seem to play a role in restrained eating. The importance of negative affect after drinking water suggests that although individuals with high scores in restrained eating may exhibit normal or even “good” cardiac and gastric interoceptive abilities and *objective physiological parameters*, the main issue concerns the emotional response to gastric visceral signals. This has important implications for interventions, as it suggests that the signal-processing per se may be intact, but that emotional response to gastric interoceptive sensations are altered. This is in line with recent studies reporting the importance of feeling of safety in one’s body and trusting bodily signals– and that especially the absence of these factors is related to ED symptoms, such as restricting or purging [53, 108]. Findings on negative affect after drinking are also in line with two studies [65, 109] according to which in individuals with AN (who display extreme levels of restricting), eating was related to negative emotions such as guilt, fear, anger and sadness. Indeed, the negative affect scale on the WLT-II [13] comprises items regarding guilt, nausea, feeling uncomfortable, sluggishness and arousal. Our results are also in line with a study [62] which found that the WLT-II drinking indices (gastric interoceptive sensitivity) did not explain dietary restraint.

In summary, the three subscales of the DEBQ were associated with *gastric attribution of interoceptive sensations* (negative affect) after the WLT-II. This is consistent with previous findings [38] suggesting that individuals practicing cognitive restraint or enforcing dietary rules may experience heightened guilt around eating, leading to a stronger emotional connection to food. This heightened emotional connection to food may render it more susceptible to influence affective states beyond mere satiety, potentially leading to the use of food as a means to modulate emotions [38]. Consequently, prolonged eating concern could alter the role of regular eating, shifting it away from improving feelings of happiness and instead associating it with negative emotionality, especially when individuals eat beyond the point of satiation [38]. Overall, our results indicate that gastric interoception may indeed be more strongly related to emotional, external and restrained eating than cardiac interoception.

### Strengths and limitations

Regarding strengths, our study used a multidimensional (*cardiac interoceptive accuracy, gastric interoceptive sensitivity, interoceptive self-report, gastric attribution of interoceptive sensations, interoceptive insight* and *objective physiological state*) and multimodal (cardiac and gastric) approach. This allows for a comprehensive examination of interoceptive processes in relation to emotional, external and restrained eating in the laboratory. Our sample comprised individuals with varying degrees of ED pathology. To our knowledge, we are the first study to use LASSO in the context of multidimensional interoception, providing a more nuanced perspective on the relationships between our variables by avoiding skewed results due to model overfit. Nevertheless, there are also limitations. First, our study comes with the usual limitations that apply to laboratory studies [110]. Some of our correlations had high *p*-values and should thus be interpreted with care. Our sample comprised a large majority of females, making it difficult to generalize to males or other sexes. Although some studies [21] suggest limited sex differences in gastric interoception and mixed findings in cardiac interoception, future studies with more balanced samples are needed. This could help clarify whether any observed effects are generalizable across sexes or if they may be influenced by sex-specific interoceptive processing differences. Interoception changes over the lifespan [111], and as our sample had a broad age-range, this may also play a role in our results. We acknowledge the limitations imposed by our sample size and emphasize the importance of replicating our findings with a larger sample to enhance the statistical power and to ensure the stability of the coefficients and the overall robustness of the model. Post-hoc power analyses indicated that the study had adequate power for detecting effects in the emotional eating model (power = 0.91). The restrained eating model was significant but underpowered (power = 0.67), suggesting that while an effect was detected, smaller effects may not have been fully captured. The external eating model was non-significant and also underpowered (power = 0.71), indicating a higher risk of Type II error. Future studies should aim for larger samples (≥ 110 participants for restrained eating, ≥ 104 for external eating) to improve power and effect size estimation. Another important limitation of our findings is that they do not predict future disordered eating behaviors or objective food consumption. We therefore suggest that longitudinal studies could be particularly beneficial in the future, for example with treatment-seeking individuals with EDs, to examine the evolution of gastric interoception alongside ED symptoms over time and during recovery. It should be noted that the DEBQ captures only certain aspects of disordered eating behaviors and is not a comprehensive measure of ED symptoms. Future research should incorporate a broader range of disordered eating behaviors, including constructs such as loss of control eating and purging, to better represent the full spectrum of eating pathology. Finally, our sample included only a small number of individuals with BN and BED and did not include individuals with AN, other restrictive-type EDs, Avoidant/Restrictive Food Intake Disorder or other ED or feeding disorders. As a result, our findings may not fully generalize to ED and feeding disorder populations where dietary restriction is more pronounced or to individuals on the lower end of the weight spectrum.

### Future research directions and clinical implications

The LASSO model demonstrated superior cross-validation performance compared to the multiple regression model, as anticipated given the large number of predictor variables. Multiple regression is susceptible to overfitting when handling high-dimensional datasets, which can compromise predictive accuracy. In contrast, LASSO’s regularization technique effectively addresses this issue, enhancing model robustness [85, 86]. Thus, we recommend considering the use of variable selection models in the context of multidimensional interoception, where the number of variables tends to quickly become large. Interoception encompasses additional dimensions [12, 32, 112], which were not included in this study and which should be integrated into future studies. Interoception is understood as not only including *accuracy, sensitivity, self-report, attribution of interoceptive sensations insight* and *objective physiological visceral states*, but also the neuronal representations, the preconscious impact of afferent signals and attention to interoception sensations [12]. Future research should therefore consider adding gastric evoked potentials [113] and the Bayesian framework (active inference) applied to gastric interoception. This may shed light on individual differences in gastrointestinal signal processing and broaden our knowledge on body-brain interactions [32].

Interventions in individuals with BN and BED may benefit from exposure to gastric signals. This is supported by our results that especially negative emotions in response to gastric signals seem to be key factors in disordered eating behaviors (emotional, external and restrained eating). Thus, future interventions may directly target these negative emotions caused by food intake and gastric activity, for example through psychoeducation or bias modification intervention. Another possibility is interoceptive exposure, which seems to be a promising approach for addressing distress and negative emotions related to food intake and gastric sensations in individuals with EDs [114–117] and should be further explored in future studies. Regarding the gastric activity part, gastric biofeedback may be another promising approach to directly target the processing of afferent physiological signals. In gastric biofeedback, gastric signals are visualized and individuals learn to modulate their gastric signals [118]. Gastric biofeedback may also be able to improve gastric interoception. Interventions such as Appetite Awareness Training (where individuals are trained to listen to their hunger and satiety cues) have shown promising results in individuals with BN [31]. Nevertheless, for any of these interventions to be effective, not only the improvement of interoceptive abilities should be targeted, but also the insight that visceral and emotional signals are trustworthy and useful [53]. One reason is that anxiety, related specifically to gastrointestinal sensations, has been found to explain disordered eating behaviors [119].

### Conclusion

In conclusion, our study explored the relationship between multidimensional cardiac and gastric interoception with disordered eating behaviors, focusing on emotional, external, and restrained eating. Cardiac and gastric interoception were found to be mostly independent. We found that gastric interoception plays a more important role in emotional, external and restrained eating than cardiac interoception. These findings highlight the importance of the gastric system in the assessment and targeted treatment of disordered eating behaviors, with trainings such as gastric biofeedback being promising intervention approaches.

## Supplementary Information

Additional file 1

## Data Availability

The datasets analysed during the current study are available in the OSF repository https://osf.io/yu9k3/?view_only=d8c03391e016413691976 da9fcc7714f .

## Abbreviations

ED: Eating disorders
BN: Bulimia nervosa
AN: Anorexia nervosa
BED: Binge-eating disorder
WLT-II: Two-step water load test
CNS: Central nervous system
MRI: Magnetic resonance imaging
HRV: Heart rate variability
HR: Heart rate
SCID: Structured clinical interview for DSM-IV axis I
DSM: Diagnostic and statistical manual of mental disorders
BMI: Body mass index
HBCT: Heartbeat counting task
ECG: Electrocardiogram
EGG: Electrogastrogram
POMP: Percent of maximum possible
IBI: Inter-beat-intervals
FFT: Fast fourier transformation
RMSSD: Root mean square of successive differences
HFnu: High frequency normalized units
NA: Negative affect
Cpm: Cycles per minute
DEBQ: Dutch eating behaviour questionnaire
VIF: Variance inflation factor
MAE: Mean absolute error
RMSE: Root mean square error
HP: Healthy participants
M: Mean
SD: Standard deviation
