# Supplementary material for: The relevance of cardiac and gastric interoception for disordered eating behavior
Source: J Eat Disord. 2025 Jun 16;13:114. doi: 10.1186/s40337-025-01284-0 (PMC12172253; doi:10.1186/s40337-025-01284-0)
Supplement: Supplementary file 1 — Additional file 1 [file 40337_2025_1284_MOESM1_ESM.docx]

**Table 7** Spearman correlations of dimensions of cardiac and gastric interoception

|  | | Sat_ml | Full_ml | Total _ml | Sat_% | Satiation | Fullness | Negative affect | Gastric interoceptive insight | Bradygastria | Normogastria | Tachygastria |
| --- | --- | --- | --- | --- | --- | --- | --- | --- | --- | --- | --- | --- |
| Cardiac interoceptive accuracy | r_s_ | .122 | .144 | .167 | -.045 | -.053 | -.074 | -.034 | .157 | -.033 | -.010 | .176 |
|  | p | .216 | .144 | .090 | .651 | .584 | .440 | .724 | .114 | .736 | .918 | .073 |
| Cardiac interoceptive sensibility | r_s_ | -.078 | **.237*** | .074 | **-.255**** | -.076 | -.040 | -.109 | -.030 | -.080 | -.030 | .110 |
|  | p | .424 | **.014** | .449 | **.008** | .422 | .672 | .245 | .758 | .415 | .760 | .259 |
| Cardiac interoceptive insight | r_s_ | .112 | **.258**** | **.200*** | -.156 | .161 | .065 | .087 | -.023 | .055 | -.099 | .015 |
|  | p | .260 | **.009** | **.043** | .117 | .093 | .499 | .368 | .820 | .581 | .319 | .879 |
| Mean HR | r_s_ | -.126 | -.064 | -.107 | -.036 | .002 | -.020 | -.023 | -.017 | .178 | .050 | -.117 |
|  | p | .197 | .515 | .275 | .718 | .983 | .836 | .808 | .865 | .067 | .607 | .232 |
| HRV(RMSSD) | r_s_ | -.058 | .023 | -.043 | -.052 | -.069 | .001 | .034 | -.030 | **-.197*** | .034 | .125 |
|  | p | .552 | .818 | .659 | .596 | .466 | .989 | .718 | .759 | **.042** | .727 | .201 |
| HRV (HFnu) | r_s_ | -.142 | -.019 | -.103 | -.065 | -.129 | -.035 | .094 | -.119 | -.025 | .053 | .000 |
|  | p | .145 | .843 | .293 | .509 | .170 | .713 | .319 | .225 | .795 | .587 | .997 |

*Note.* **Full_ml** = additional water volumes ingested until maximum fullness.  **HFnu** = high frequency normalised units.  **Mean HR** = Mean Heart Rate.  **RMSSD** = root mean square of successive differences. **Sat_%** = percentage of satiation to maximum fullness. **Sat_ml** = mean volume of water ingested until satiation. **Total_ml** = total water volumes ingested

**Table 8** Spearman correlations of dimensions of gastric interoception with disordered eating and BMI

|  | | Sat_ml (*n* = 106) | Full_ml (*n* = 106) | Total _ml (*n* = 106) | Sat_% (*n* = 106) | Satiation (*n* = 114) | Fullness (*n* = 114) | Negative affect (*n* = 114) | Gastric interoceptive insight (*n* = 105) | Brady gastria (*n* = 106) | Normo gastria (*n* = 106) | Tachy gastria  (*n* = 106) |
| --- | --- | --- | --- | --- | --- | --- | --- | --- | --- | --- | --- | --- |
| DEBQ emotional | r_s_ | **.192^*^** | 0.101 | .181 | .064 | **.315^**^** | **.299^**^** | **.350^**^** | .061 | .157 | **-.237^*^** | .052 |
|  | p | **.049** | .305 | .064 | .517 | **.001** | **.001** | **.000** | .539 | .108 | **.015** | .598 |
| DEBQ external | r_s_ | **.193^*^** | .009 | .121 | .136 | .093 | **.197^*^** | **.314^**^** | .057 | .116 | .049 | -.187 |
|  | p | **.048** | .930 | .216 | .165 | .324 | **.036** | **.001** | .567 | .238 | .617 | .055 |
| DEBQ restrained | r_s_ | .112 | .066 | .110 | .019 | .093 | .100 | **.301^**^** | .104 | .025 | .008 | .040 |
|  | p | .255 | .500 | .262 | .849 | .323 | .289 | **.001** | .293 | .802 | .937 | .686 |
| BMI | r_s_ | .072 | .075 | .079 | .038 | .066 | **.191*** | .110 | -.173 | .086 | **-.229*** | .107 |
|  | p | .460 | .442 | .422 | .699 | .485 | **.042** | .248 | .077 | .386 | **.019** | .277 |
|  | | | | | | | | | | | | |

*Note.* **BMI** = Body Mass Index. **DEBQ** = Dutch Eating Behaviour Questionnaire (van Strien et al., 1986). **Full_ml** = additional water volumes ingested until maximum fullness. **Total_ml** = total water volumes ingested, **Sat_%** = percentage of satiation to maximum fullness. **Sat_ml** = mean volume of water ingested until satiation.

**Table 9** Spearman correlations of dimensions of cardiac interoception with disordered eating and BMI

|  | | Cardiac interoceptive accuracy (*n* = 110 ) | Cardiac interoceptive sensibility (*n* = 114 ) | Cardiac interoceptive insight (*n* = 109 ) | Mean HR (n = 113) | HRV(RMSSD) (n = 113) | HRV (HFnu) (n = 113) | DEBQ Emotional (*n* = 120) | DEBQ external (*n* = 120) | DEBQ restrained (*n* = 120) |
| --- | --- | --- | --- | --- | --- | --- | --- | --- | --- | --- |
| DEBQ emotional | r_s_ | -.047 | .054 | .125 | -.136 | .080 | -.125 | 1 |  |  |
|  | p | .623 | .569 | .194 | .151 | .397 | .188 |  |  |  |
| DEBQ external | r_s_ | .039 | .071 | .096 | .020 | .000 | -.085 | **.507^**^** | 1 |  |
|  | p | .684 | .453 | .321 | .834 | .999 | .371 | **.000** |  |  |
| DEBQ restrained | r_s_ | .150 | .049 | .100 | **-.193^*^** | **.226^*^** | .104 | **.353^**^** | **.206^*^** | 1 |
|  | p | .118 | .602 | .300 | **.040** | **.016** | .272 | **.000** | **.024** |  |
| BMI | r_s_ | -.136 | .034 | -.007 | .020 | -.103 | .016 | **.346**** | **.200*** | **.196*** |
|  | p | .159 | .722 | .943 | .831 | .281 | .863 | **.000** | **.032** | **.035** |

Note. **DEBQ** = Dutch Eating Behaviour Questionnaire (van Strien et al., 1986). **HFnu** = high frequency normalised units. **Mean HR** = Mean Heart Rate. **RMSSD** = root mean square of successive differences.
